# The Meaning of Social Participation for Daily Mobility in Later Life: an Ethnographic Case Study of a Senior Project in a Swedish Urban Neighbourhood

**DOI:** 10.1007/s12126-017-9296-4

**Published:** 2017-06-05

**Authors:** Vanessa Stjernborg

**Affiliations:** 1 Department of Urban Studies, Faculty of Culture and Society, Malmö University, -205 06 Malmö, SE Sweden; 2 K2 – The Swedish Knowledge Centre for Public Transport, Scheelevägen 2, 223 81 Lund, SE Sweden

**Keywords:** Place, Context, Older people, Wellbeing

## Abstract

This paper presents an ethnographic case study that aims to understand the meaning of social participation in a neighbourhood for daily mobility in later life. In the study, the mobility of the participants of a senior-citizen project was monitored over 18 months. The project was founded as a result of a municipal district’s targeting of social sustainability. The results show that social participation had positive effects on the daily mobility of the participants. The implementation of broad-minded thinking from the municipality and the cooperation of various municipal actors were shown to be essential for the positive outcome of this project.

## Introduction

Mobility is considered essential in our everyday lives, especially for social participation and managing everyday activities (Levasseur et al. 2015; Hjorthol 2013; Schwanen and Ziegler 2011; Ziegler and Schwanen 2011; Mollenkopf et al. 2004a, b); and it is central in enabling older people to participate in society (Zeitler et al. 2012; Mollenkopf et al. 2004a; Mollenkopf et al. 2004b). Moreover, it is often the case that mobility is associated with independence and freedom, while a lack of mobility can give rise to feelings of social deprivation and exclusion (Urry 2007).

Mobility and social participation are two important determinants for wellbeing (Nordbakke and Schwanen 2014; Schwanen and Ziegler 2011; Ziegler and Schwanen 2011; Dupuis-Blanchard et al. 2009; Mendes de Leon 2005; Metz 2003; WHO 2002; Metz 2000) and health in later life as well as for successful ageing (Levasseur et al. 2015; Rowe and Kahn 1997). The Universal Declaration of Human Rights considers mobility and the right to move independently as an important part of human rights (UN 2016). However, mobility is more than simply moving from point A to point B (Cresswell 2006); rather, it is a combination of the physical, social and mental capacities for movement (Zeitler et al. 2012; Schwanen and Ziegler 2011; Ziegler and Schwanen 2011; Metz 2000). Thus, mobility is influenced by the context within which the individual is embedded and interacting, such as the community, the household, the family and the wider society (Hanson 2010). In a comprehensive synthesis of empirical studies on the association of neighbourhood environments with mobility and social participation in later life conducted by Levasseur et al. (2015), the researchers emphasise the importance of examining the combination of both mobility and social participation in future studies.

However, barriers to movement can occur in different forms and shapes, and they can result in negative social impacts in different ways (James et al. 2005). They can be physical barriers that hinder the mobility of the person, for example, traffic infrastructure with wide roads and heavy traffic, or they can be psychological or perceived, such as with individuals who are disturbed by such barriers as traffic noise, road safety fears, the fear of crime, and so on (Risser et al. 2010; Wennberg et al. 2010; Wennberg et al. 2009; Ståhl et al. 2008). Other factors that can contribute to a negative effect on the possibilities for movement are low social capital, reduced health and lower functional capacities (Tuan [1977] Tuan 2011; Rosenkvist et al. 2010). Moreover, age, gender and class-related factors can have an influence (Ziegler 2012).

Interest in the meaning of social participation in a community in later life is growing (WHO 2015). Social participation has been identified as a key strategy for fostering empowerment and engagement, and it appears particularly beneficial for older people, as they are often associated with a range of health indicators (Richard et al. 2009). By studying definitions of social participation published between 1980 and 2009, Levasseur et al. conclude that, overall, the definitions “mostly focused on the person’s (who) involvement (how) in activities that provide interactions (what) with others (with whom) in society or the community (where)” (Ibid: 2144). It is from this standpoint that the concept of *social participation* is used in this paper.

Research indicates that persons with high levels of social capital often tend to interact more in their community, for instance, by volunteering, by having political commitments, and by socialising more frequently with friends and neighbours (Leyden 2003). In turn, this can reduce feelings of fear (De Donder et al. 2005) and can be a possible key determinant for successful and healthy ageing (Levasseur et al. 2010). In other cases, researchers have emphasised the role of place of residence and issues of social exclusion and inclusion (Scharf et al. 2002a; Scharf et al. 2002b; Scharf et al. 2005; Phillipson 2007). Older people in underprivileged neighbourhoods are often regarded as being particularly vulnerable (Buffel et al. 2013; Smith 2009; Scheidt and Windley 2006).

Different spaces and neighbourhoods offer different conditions for social participation (Lager et al. 2015) and mobility in later life (Föbker and Grotz 2006). Although this is not necessarily always the case, ageing in an urban context can create environmental stress in the form of poor housing conditions, crime-related anxiety, feelings of fear, and social polarisation and exclusion (Buffel et al. 2013; Rodwin and Gusmano 2006). Another issue is the role of changing landscapes, with increasing urban segregation and growing income inequalities being highly relevant for Swedish cities as well. According to Ziegler (2012), the nature of social interaction in neighbourhoods has been affected by societal and structural changes where “decreasing trust and changing norms have led to a physical and social distancing between generations and neighbours” (Ziegler 2012:1302).

In this paper, the home neighbourhood is a central part of the context that the older person is situated in. According to a highly-informed review of ageing and mobility, more context-dependent research of mobility in later life is needed (Schwanen and Páez 2010), where a vital factor is the place of residence in relation to social participation and daily mobility.

With this background, I would like to highlight the mobility of older persons as place- and context-dependent. The introduction focuses on the relation of mobility and social participation and on the importance of place for daily mobility. I also want to highlight the role communities play in the important work of supporting mobility in later life and include this group in the continuing work for both neighbourhood development and more sustainable communities. This paper aims to provide an understanding of the meaning of social participation in a neighbourhood for daily mobility in later life. As a researcher, I followed a group of seniors for 18 months in an underprivileged neighbourhood located in Malmö in the south of Sweden. The group is part of a municipal project that aims to strengthen networking and the idea of social participation among senior citizens. This reflects a societal effort to achieve social sustainability through daily mobility and social participation carried out by the implementation of social activities. This study can also serve as a limited project evaluation in terms of process and impact.

The introduction of the paper is an outline discussion of key elements of the study, namely, social participation and daily mobility in a neighbourhood in later life. Thereafter, a description of the study district and the senior project in the neighbourhood is presented. This is followed by a description of the method used. Then, research findings are presented in two parts – the first describes the senior project in detail, while the second provides an understanding of the meaning of social participation for daily mobility in later life. Finally, the discussion concludes the paper.

## Study District – The Neighbourhood of Seved

Previous research often addresses ageing and everyday life on a macro scale; alternatively, it focuses exclusively on areas dominated by older residents, which makes studies on other types of neighbourhoods especially important. Therefore, in Sweden, the needs of and the challenges faced by older people in underprivileged districts is an overlooked research area.

The district of this study is Seved: a so-called socioeconomically poor inner-city neighbourhood located in Malmö in southern Sweden. Malmö is Sweden’s third largest city, with just over 328,000 inhabitants as of 2016. It is a segregated city, with both ethnic and economic residential segregation (Hedin et al. 2012), and a total of 44% of the population has a foreign background (either born outside of the country or with both parents born outside of the country) (Malmö 2016). There are also large differences in the average income of the different urban districts. All of which lead to another image of Malmö: one of a dynamic city focused on innovation and knowledge – a so-called knowledge city (Mukhtar-Landgren 2005). Malmö has been a successful city in many ways, for instance, concerning ecological sustainability. It is sometimes even referred to as the “city of tomorrow” and has gained recognition for best practice (Pitts 2004). However, the city still faces several challenges regarding social sustainability.

The area of Seved, which serves as the study district, has a population of approximately 4500 inhabitants (approximately 350 of them are 65 years and older). Partly due to its central location and vicinity to the city centre, as well as it close proximity to services and transport modes, Seved has recently attracted new and young residents. The population is already quite young, with half of the inhabitants aged between 15 and 39 years. Seved is also socioeconomically vulnerable and is often negatively portrayed in local news and on national television (see Stjernborg et al. 2015). More than 60% of the population has a foreign background, and the average income is substantially lower (almost a third lower) than the average income for Malmö as a whole.

## The Senior Project

In 2010, the municipality launched the ambitious *Area Programmes* for a more socially sustainable city. Several districts and neighbourhoods were targeted in this programme, including the neighbourhood of this study, Seved. The overall aim of the programme was to foster and strengthen local democracy and empower citizens’ participation in the affairs of their township. As part of this programme, a seniors group was established with the intention of strengthening the social networks of seniors, of preventing isolation and implicitly supporting social participation and daily mobility in the neighbourhood in later life.

One aim of the senior group was to strengthen the seniors’ social network and to increase their notions of social participation in later life. Daily mobility was initially mostly actualised through a thematic meeting that included a coffee klatch in the neighbourhood meeting-place,^1^ which was centrally located in the district. Invited guests from NGOs and public health and welfare groups raised ageing issues and gave advice on nutrition and fall prevention. This indicates that the primary action tended to focus on physical health.

However, during spring 2012, and inspired by local initiatives concerning children’s activities, the activities expanded to include those outside the neighbourhood. Multidimensional, cross-sector activities for children and youth spilled over into the previous social sector-related activities for adults and older adults. In addition, cultural facets were added: an adult educational association, the city museum, the city theatre and the city archives joined in co-operation, which was unique from a Swedish perspective. During the same year, around 20 activities were arranged that included around 40 seniors. The goal for the coming years was to frame the activities in close co-operation between the seniors and the working group in order to combat social isolation among the seniors in the area: the aim was to collect the life histories of the older persons, reach more seniors in the neighbourhood, identify the needs of the seniors, and develop new ideas and implement activities for seniors in Seved, all of which would continue even after the Area Programmes terminated in 2015. The activities were free for seniors and financed through cross-sector co-operation; for instance, a museum sponsored some of the activities and two teachers, and a theatre provided theatre tickets and coffee. In addition, a local municipal real estate company supported some activities, while the city council (social welfare and health departments) provided a limited budget and undertook responsibility for the programme. This meant a small budget was available to cover transport costs, coffee klatches, and so on. Over the duration of the study, the senior group frequently grew in number and new municipal actors were willing to participate in the project.

## Method

### The Ethnographic Method

This paper is based on an ethnographic case that studied the daily mobility and social participation of older persons in a neighbourhood. The ethnographic method is especially suitable for contextually studying the daily lives and cultures of human agents (O’Reilly 2005), which means studying the micro-world within and with reference to a macro-world (Marcus 1998). The method enables the researcher to collect valid and reliable data through ongoing and close contact with those being studied; the method is an established fieldwork method (Gold 1997).

### Data Collection

The ethnographic material collection began in December 2011 and lasted until April 2013. As mentioned, during 2012 around 20 activities were arranged and included around 40 seniors in total. However, the number of participants varied from activity to activity. The empirical collection ended after saturation, when no additional material emerged and similar instances were found to be consistently occurring (see Glaser and Strauss 1967). The empirical material consists of participant observations (including conversations and field notes) and interviews (seven older persons and one programme worker), all with the purpose of collecting observations, experiences and reflections; therefore, it was a *substantive sampling* (Gold 1997).

With a substantive sampling approach, the objective is to generate exhaustive empirical accounts of how people perceive, experience and make sense of their daily situations (ibid.). During the study period, I attended all planning and workgroup meetings, joined programme activities and travelled on all arranged activities outside of the neighbourhood. The community arranged transport for all the activities outside of the neighbourhood, either rented busses or taxi cabs, depending on the number of participants from the neighbourhood, of which I was included in a research capacity. All in all, this resulted in site visits a couple of times per month; consequently, I became a known face among the seniors and the staff. The empirical material was collected through *first-hand* involvement, with the purpose of studying the participants *in action* (Murchison 2010:4). Prior to commencement, the study was approved by the Regional Ethics Board in Lund (file number 2011/265).

### Field Notes and Interviews

Field notes were carefully collected in a field diary, directly and in connection with each meeting or activity. After participating in meetings and activities for almost one year, interviews were conducted. The participants were asked during some of the activities if they would be willing to participate, and seven participants (six women and one man between the ages of 60 and 85) accepted. The gender bias reflects both the difference in life expectancy between men and women, in general, and the relatively higher percentage of older women in this neighbourhood. One reason given for declining to be interviewed was “interview fatigue”, meaning that they felt too many people were asking them too many questions on too many subjects far too often. However, when asked to be more specific, the participants said that they regarded the interviews as similar to dialogues and conversations with doctors and occupational therapists. The interviews were complemented by the many observations and conversations between the seniors and the workers that took place during arranged activities and meetings, where reflections, opinions and experiences were all recorded in the field diary.

The interview format was semi-structured, covering different aspects of social participation and mobility, such as daily activities, modes of transport in daily life, and social activities and daily mobility in the neighbourhood. Themes addressed during the staff interviews contained mostly processes, and they mirrored the perspectives of the participants. All interviews were recorded and transcribed.

Validation of the empirical material was made throughout the study period by checking with informants to ensure that there were no misunderstandings or misclassifications (see Gold 1997). For the analysis, multiple readings were made of the material from the field diary as well as from the transcribed interviews.

To gain knowledge of the content of the collected empirical material, a number of readings were made. The empirical material was initially organised into categories of social participation and mobility before a more nuanced analysis could be conducted. Through following a line-by-line reading, a list of themes emerged, including more specific issues of daily mobility, social context and social relations in the neighbourhood, as well as more detailed issues of the senior project. The collected material was then coded according to these themes. Consequently, the chosen ethnographic method is essentially inductive. Due to ethical reasons, statements in the following text have been anonymised and names are fictitious.

### Social Participation Programmes

The senior project in the neighbourhood of Seved is, as mentioned, an initiative launched by the municipality to prevent isolation and implicitly support social participation and daily mobility in the neighbourhood in later life. Through an evaluation of Raymond et al. (2013) of social participation programmes, some important conditions emerged, even though the evaluation shows that it is difficult to create a list of best practices. Three conditions relate to the lifestyle, identity and agency of seniors, while another three conditions refer to the organisational and structural aspects of the programme.

Briefly, the recommended conditions concern:

- Recruiting participants in their own living environment.
- Providing activities that acknowledge and respect the interests, needs, experiences and cultures of the older persons.
- Supporting the development of meaningful social relationships and roles.
- Participation in the planning, realisation and evaluation of programmes and being a part of the decision-making process.
- Training staff for practices that favour democratic and participative management.
- The need for a sufficient programme duration (a minimum of six months) to foster a sense of belonging and the development of social relationships (Ibid: 289–290).

The conditions presented above will be of importance for the forthcoming analysis. In the following section, ethnographic narratives from the field notes and from the interviews are presented.

## Ethnographic Narratives

This section is divided into two parts. The first describes the senior project in detail from a more organisational perspective with a focus on processes and impacts, while the second describes the context, the social participation and the daily mobility of the senior project participants with a special focus on the meaning of social participation in a neighbourhood for daily mobility in later life.

### The Senior Project in Detail

A summary is presented in Table 1, where overarching results during the study period are divided into two clusters – processes and impacts – of the senior project in the neighbourhood of Seved. The table aims to offer a more easily accessible overview for the reader; also, the processes and impacts are more closely described in the following chapter.

**Table 1:** Processes and impacts of the senior project evaluation in Seved

Process	Impact
The establishment of a working group – leaders on different levels from the local city councils (social welfare and health), with one representative from the local health care centre and one senior organisation representative.	‘Crossing borders’ – seniors from other parts of the city participate in the many activities offered in the neighbourhood of Seved.
Initial priority – social sustainability, security and social participation among older people with a special focus on wellbeing.	The ‘meeting place’ has become important in the daily life of the seniors – even if it’s just having a cup of coffee and someone to talk with.
Key issue: How can we work in a slightly different (better) way, given our (limited) resources?	Increasing effects on the wellbeing of senior participants.
Initial activities – thematic meetings with guests from NGOs and public health/welfare, etc. The embracing of a wide perspective, involving other contacts like libraries, associations, and the local real estate company that sponsored a venue, etc.	More closed-off societies – where risk of isolation and immobility increases. The senior group is important for social participation and networking among people that otherwise could be at risk of loneliness and exclusion.
Challenges: How to reach out with information? How could the negative image of the municipality be changed? Elaborated solutions: focusing on visibility and presence in Seved; advertising in a monthly letter; meeting-place door advertisement; spreading through word-of-mouth from home care service staff, senior neighbours, etc.	Taking advantage of accessible resources, including the older people, to increase the social participation in the neighbourhood has had a positive effect on the daily mobility of senior participants as well as increased the social participation among those people.
Multidimensional, cross-sector activities for children and youths spilled over into social sector-related activities for older adults. Cultural facets were added: one adult educational association, the city museum, the city theatre and the city archives joined in co-operation, and the network always continues to grow.	Members of the senior group are more visible in the neighbourhood in daily life. Increase in mobility mostly involves walks within the neighbourhood with friends from the senior group, walks to the meeting place from home, and trips outside the neighbourhood through arranged activities.
Goals: Frame activities in close co-operation between senior participants and the working group to combat isolation among older people in the area; collect the life histories of the older persons; reach more seniors in the neighbourhood; determine needs and develop new ideas; and implement activities for older people in the area that will remain even after the Area Programmes end in 2015.	The participation has led to reduced feelings of fear for some and more social meetings between older persons and youths in the neighbourhood.
Free activities for senior participants, financed through cross-sector co-operation.	Attachment to place – descriptions of how lucky they are, living in a ward that does so much for them in their daily lives.
The policy of stakeholders – based on an interdisciplinary mind set and inclusion – is an important issue. Cross-cultural actions to foster meetings across cultures and ages, like the invitation of pre-schools, etc.	Spin-offs: Senior participants have been inspired to take their own initiatives for activities – e.g. a walking group with walking poles (“Nordic walking”) and “sitting gymnastics”, both led by the senior participants themselves.
A mix of activities – thematic meetings, etc., within the neighbourhood, and social and cultural activities outside the neighbourhood.	The social context is important. Even if, in theory, they have sufficient mobility resources, the senior participants assume that they would not do some of the activities in practice.

At the outset of this study in late 2011, the senior working group had existed for a year. A key priority was solving the issue of “How can we work in a slightly different (better) way, given our (limited) resources?” Other priorities stressed by the working group were social sustainability, security and social participation among seniors, with a special focus on wellbeing. The working group consisted of leaders of different levels from the local city council (social welfare and health department), that is, one representative from the local health care centre and one senior organisation representative. Thematic senior meetings were already established, and discussions revolved around whether or not activities should be expanded. Bingo meetings were also already established as an initiative from the seniors themselves, with meetings once a week as a result of an earlier project. The aim of the working group, which was very open-minded, was to find new ways (with small contributions) to improve output.

One challenge identified early on by the working group was how to reach out with information. Based on previous experience, brochures and papers from the municipality were disregarded. A question posed from the working group was; how could the negative image of municipality initiatives be changed? It was agreed that workers from the municipality would like to be more visible in Seved. By focusing on visibility and presence through advertising in a monthly letter to all residents in the neighbourhood and through door-to-door advertising, the initiative for senior citizens gained attention. However, the wide dissemination of the activities through word of mouth also had an impact: from home care service staff or other activities related to the senior project, and also between senior neighbours themselves. The arrival of new participants was noticeable; often, they would be friends or neighbours of the other participants. The policy of stakeholders engaging in senior activities was based on an interdisciplinary mindset. Another important issue was inclusion. For example, cross-cultural and intergenerational actions to foster meetings across cultures and ages were also implemented later. A case in point was the invitation to pre-schools to join some of the activities to create meeting places that transcended generations. Many of the children who participated were either first or second-generation residents. This cross-cultural and intergenerational work proved to be successful; consequently, more shared activities were on the agenda.

Initially, the aim was to take a wide perspective and involve other contacts, such as libraries and other associations, for the thematic meetings. To a great extent, the activities came to revolve around historical time periods – “now” and “then” – realised through a line of innovative activities, such as visits to museums; theatres; guided city tours; Christmas fairs; barbeques on the beach; forest walks; lunch at the top of Malmö’s second tallest building; and a demonstration of how herring are cleaned, followed by a boat tour on the canal, and so on. Another promotional activity was the making of ‘calendar seniors’, with pictures of the Seved seniors in historical costumes from the city museum. Involving seniors in the planning phase proved to be a crucial factor in ensuring their satisfaction with the arrangement. Throughout the entire process, suggestions, reflections and participation from the seniors were important; moreover, the seniors were treated with democratic values and humility. By viewing the senior project in a wider perspective, it is much in line with Raymond et al. (2013) earlier-mentioned conditions.

The local real estate company also sponsored events for the social engagement of the seniors in the neighbourhood by, for example, allowing access to a venue for the thematic meetings. The strategy made it possible for older persons to age in place, and it consisted of three goals: preventing isolation, making housing accessible and providing home safety packages. According to the real estate company representatives, much of their work is based on making it possible for people to enjoy their neighbourhood so that they would remain as residents.

### The Meaning of Social Participation in one’s Neighbourhood for Daily Mobility in Later Life

Overall, the average age of the participants was quite high, with many being close to 80 and over; a few were even over 90. Most of the participants had children, though not all. Many had worked in places like the post office, bakery, haberdashery “shops”, home care services, the railway station, and on the road as truck drivers, to name a few. In daily life, the older people spoken to seemed to be overall satisfied with the available modes of transport. Further, the reasons they mentioned for sometimes avoiding doing things outside of the home were more of a social character and not because of insufficient access to means of transportation. Not one drove a car, or even owned a car anymore, for that matter. Several had a driving licence and had owned a car earlier in life, but others never had a licence; consequently, they always used alternative modes of transportation, such as public transport and/or a bicycle. None of the older people interviewed missed having a car, and, overall, they were satisfied with the transport options at hand. There were few reasons that necessitated driving, but, on the whole, there was no need as they lived in a central district and car parking was too expensive for just “driving for pleasure”, to use one of the older person’s reasoning.

Many of the senior activity participants had lived in their apartments (all interviewees live in apartment buildings) for many years; a few had resided there all their lives, while others for at least 40 to 50 years. A few had moved from apartment to apartment within the neighbourhood, while others had moved within the central areas of the city. A common factor shared by all was having experienced central urban living for many years, which entailed close proximity to services and public transport. A few of the participants were born and raised in the countryside but had moved to the city as young men and women. Moreover, a few of the participants lived outside Seved, but still in central parts of the city. Some reasons for ‘crossing borders’ and participating were that they had friends living in Seved who had told them about the many enjoyable activities taking place there and also of the people involved. Both outsiders with no obvious links to Seved and those with previous close familiarity with the place were, through their involvement in the senior activities, able to create a meaning of the place in a positive way.

Overall, a difference regarding household structures was noticed amongst the participants, with singles more active in various activities than those living in two-person households. One widow related how she had been quite busy during her husband’s ten years of illness. During that period, he was living in a home for the elderly, where she visited him every evening, watching television with him, and so on. A consequence of her spouse living away from home for so long was that she adapted to being alone. Therefore, she was prepared for his passing, which happened a couple of years ago. In fact, his passing was a relief, as she was then able to focus on herself. At the time of the study, she was over 80 years old and led a very active life, taking part in various daily activities with the other senior participants. Another widow also informed that she began participating in neighbourhood activities when her husband passed away and that she became more social with her neighbours after his death:

Before, when I was living with my husband and that, I was never down in the common garden and that. But then I saw them sitting down there, and it looked very nice and that. So, I talked to them, and so on. But it was not until last year they said, “But come down”, and I said, “It looks so nice”; and they said, “Yes, so come down”. That was what they said, those who often sit down there; and in that way, we got acquainted in another way.

One woman in a two-person household described how she often felt very lonely and how her ageing husband did not want to talk to her very much anymore. She explained that she more or less felt lonely her whole life, being an only child with no children of her own. For her, the neighbourhood meeting-place was important, especially because of a man who worked there:

I cannot speak with my husband, and, therefore, I have to go down to Adam^2^ to speak with him.

Both the older persons and those working within, or close to, the project regarded the senior activities as very successful. It was apparent that the staff were proud to work with the project and that the interest they showed in the various measures and outcomes was genuine. One man, in particular, stood out as an important actor; indeed, he was regarded and acknowledged as a true driving force by many of the older persons. All the seniors had formed a close attachment to him and frequently dropped by the meeting place to see him while out on daily errands and such.

For many of the older persons, participation in the neighbourhood was an important aspect of their daily mobility. As several had health problems, they used walking aids to get around; others were relatively healthy and mobile. Notwithstanding the foregoing, they were all quite active. Indeed, those I spoke to reported that they – as part of a social network – strove to leave their home every day.

For the seniors, social context and ambience were crucial not only for facilitating their mobility but also for drawing them out of their homes. Many of them repeatedly described how lucky they were to live in an area that did so much for them in their daily lives. The activities resulted in a supportive network that continued to grow organically. Because of the project, the seniors were inspired to take the initiative to start other activities. For example, they formed a walking group, which walked around the neighbourhood once a week with walking poles (“Nordic walking”). Another example was a very popular senior-led group that did “chair-based exercises” once a week.

Both programme activities and consequent spin-offs were clearly important for participants as they helped fill their lives with meaning, thereby supporting wellbeing. One participant over 90 related how lonely she used to be. Over the years, her social network eroded, leaving her unacquainted with her neighbours. Moreover, her daughter had passed away just a couple of years previously, causing her depression and grief. However, since participating in the programme activities, her life was filled with content and meaning. She then had friends and acquaintances, and saw herself within a social context and as belonging to a supportive network. Several of the senior participants mentioned the value of having someone who called just to check how they were doing, which can be seen as a consequence of having made new contacts and friends through the senior project.

All the participants had experienced changes – major or minor – in their daily lives since the launching of the programme. Not only were the activities related to current issues, but they also embraced the fact that participants had lived through a period of time of dramatic change, that is, moving from rural life long ago to the city, which was in post-industrial transition. One participant, who was over 90-years old, suddenly started crying during one of the activities, a bus excursion to a garden and flower store just outside of Malmö. Her eyes filled with tears as she looked at the cornfields from the bus window. Though she was born on a farm, she had not had the opportunity to see the countryside for many years.

Small talk with the seniors during the activities (theatre, museums, various outdoor activities, and so on) often revolved around their gratitude and appreciation of being part of the senior group. They stressed that they would never had done such things on their own, or as one woman expressed it, “That is something that you don’t do by yourself. It isn’t fun”. It was the social context that was important. So even if, in theory, they had sufficient mobility resources, they assumed that they would not do similar activities by themselves in practice. Again, we see that mobility is more than the physical movement from point A to point B:

I think they are doing a nice job, both the municipality, Maria, and the ones with Christmas coffee, and then we have the celebration around Midsummer, and we are sitting outside. And everything they arrange, what Adam and they arrange, and it is… yes, we went to the theatre, too. If it weren’t be for them doing all this for us, we would just be sitting there [at home].

The seniors talked about the activities as the opposite of being alone: “You don’t want to be sitting alone”, or “It is boring being at home alone. You need company”. Some of the participants would not do the activities (or similar activities) by themselves, even if they easily could have. Part, or even a substantial part, of the joy came from the collective action: going to the theatre alone, or even with a friend, would not have been as enjoyable and meaningful. Several of the older persons stated that they left their home more often simply because of the senior group. They had a network of people surrounding them, friends as well as programme staff in the area, which resulted in them not feeling as lonely and isolated as they had before.

The meaning of the work from the municipality became very clear, according to one of the participants. Through rehabilitation, this person, who had been ill, had come a long way towards a healthier and more active lifestyle. An attendant from the municipality visited him once a week and sometimes accompanied him for long walks and on public transport. He credited the assistance of the attendant for his recovery and that he was able to live a normal life again. For him, resources from the municipality and social relations from a friend were of the utmost importance for his recovery to normal life. He explained that he had thought of giving up many times. However, the attendant and others involved gave him the tools to carry on and strengthened his determination. Although still struggling with deteriorating health, he was at that time highly active, often visiting the meeting place and joining most arranged activities. He communicated that his life had renewed meaning because of social participation:

Then I was allowed to go out to the staircase, and one stair up and one stair down. It took me three weeks before I came down to the common garden. They trained me for seven months […]. Then I had my attendant. She came on Mondays, and then I just sat there waiting for her to come on the next Monday too. I just sat there, angry with myself, and then I said to myself, “You! […]” And then I went out. I took my walker and walked to Sevedsplan [the neighbourhood square where the meeting place is located]. I knew the way, so then I could walk longer and longer, but I only walked the ways I knew.

The man related how he often walked to the meeting place for a cup of coffee or to play some table tennis with one of the workers and how today he is well-known in the area. He seemed attached to the place, and walking on familiar streets also appeared important to him during his recovery. In addition, he also partook in most activities arranged outside of the neighbourhood.

Another issue brought up by seniors and workers was the role of changing landscapes and the ‘social transformation’ of neighbourhoods. Several of the participants mentioned a transformation where socialising with neighbours had changed over time. Today’s social landscapes are changing in many ways. Growing gaps and social segregation with increased surveillance resulting in more closed communities are becoming more common. Other reasons for the lack of socialising, according to some senior participants, was that younger people today are busy with their careers, working long hours, and they do not have the same amount of time for socialising they had when they were younger. Regarding neighbours and neighbour relations, most of them agreed that Seved has changed. More young people are moving in, and they tend not to socialise in ways that were customary in the old days:

We had very nice times down there (in the common garden). We had long tables and a barbeque and … but it is not like that anymore, and it will never be like that again because … those youths, they do not stick together. They could go down there and have a barbeque, but they don’t. So that won’t be [happening] anymore.

There is a risk that isolation and immobility will increase, with more closed-off communities and less socialising between neighbours among other things. Through the field studies, it became clear that the municipality makes an important contribution by increasing the social participation and social networking of the seniors, who would otherwise be at risk of loneliness and exclusion.

To summarise, according to the participating seniors and those working with the project, the mobility of the former increased because of the changed social context. Through various descriptions of the meaningful activities, this also appears to be closely connected to greater wellbeing. The only fear some of the seniors expressed is that the project would likely run out of funding at some future point. Moreover, most of the senior group acknowledged the benefits of being out more and having increased mobility. This is a result of the larger social network and more daily activities, involving walks in the neighbourhood with friends from the senior group, walks to the meeting place from home, and trips outside the neighbourhood through arranged activities.

## Discussion

This study has shown why it is important for communities to work with social participation to support mobility in later life and to include senior citizens in the continuing work for neighbourhood development and for more sustainable communities. It has highlighted the importance of creative and user-centred campaigns for the social participation and daily mobility of older persons. The implementation of broad-minded ideas from actors working with these issues were shown to be crucial; in this case, the key issue for the municipality was solving “how can we work in a slightly different (better) way, given our (limited) resources?” with initial priority on social sustainability, security and social participation, and wellbeing among older people. The result of proceeding from this basic question and the initial priority is a unique form of cooperation from a Swedish perspective, with contributions from various municipal actors. However, the networking and the cooperation was an ongoing process. The cooperation was resource-effective, despite not having a large budget from any one source.

The project proved to be successful in different ways, and it led to increased social participation and networking, and increased daily mobility among senior participants. The dispersion effects in the form of new activities arranged by older people, for older people, were additional positive results. The project also contributed to older people becoming more visible in the neighbourhood. In an evaluation of social participation programmes by Raymond et al. (2013), some important conditions emerged; as mentioned earlier; these conditions were fulfilled in this study case. The participants were recruited in their own living environment; the activities both respected and acknowledged the interests, experiences and cultures of the older persons involved; the project supported the development of meaningful social relationships and roles; the senior participants were a part of the planning, realisation and evaluation of the programme as well as a part of the decision-making process; the staff supported a democratic and participative management; and, finally, the project lasted for longer than six month (see ibid. 289–290).

From a wider perspective, it is important not to overlook the perspectives and impact of ageing, and the needs of older persons in urban neighbourhoods; not least for the continuous work to prevent the development of growing gaps, segregation and deprived neighbourhoods. A ‘social transformation’ of the neighbourhood was visible in the study were, for example, socialising with neighbours had decreased over time. It was mostly women talking about this transformation, which probably can be related to the gender roles that this generation has grown up with. Traditionally, women were, generally, not expected to socialise or to be independently mobile outside of the family circle or the own neighbourhood (Ziegler 2012), which makes social relations with neighbours even more important. Conclusively, the project facilitated new ways to create social relations in the neighbourhood.

Other issues concerned the neighbourhood compound and the mixture of people of different ages and with different backgrounds; for this reason, cross-cultural and intergenerational actions to foster meetings across cultures and ages were implemented. These meetings were, according to the working group, very beneficial for all involved and important in the ongoing work with bridging the gaps between people of different generations and different nationalities.

The enhanced knowledge of older people’s perspectives and roles is important, not least when starting target programmes for social sustainability, for example, in the restructuring or refurbishing of deprived housing and the revitalising of neglected outdoor environments. Stakeholders in Malmö have realised the value of social sustainability among older persons. Consequently, these issues are a high priority for the local city councils (in terms of social welfare, as well as health divisions) and also for economically sustainable developments by housing companies. Focusing on social issues, amongst others, is necessary for preventing social isolation and reducing mobility among certain groups and individuals.

As a result, it is important to recognise the daily mobility of older persons as place- and context-dependent and essential for social participation and for managing everyday activities. The individuals and their lives are socially situated agents within place and context. In addition, mobility means more than physical movement; it also involves physical, social and mental capacities (Zeitler et al. 2012; Schwanen and Ziegler 2011; Ziegler and Schwanen; Metz 2000). This has been illustrated in this paper in different ways. The results shows, for example, how challenges related to low physical capacities can be overcome through social participation. Regardless of functional capacity and age (many were over 80 years old), the senior participants were all quite active and strived to leave their homes on a daily basis. Urry (2007) associate the ability to be mobile with independence and freedom, and several researchers highlight the importance of mobility and social participation for wellbeing. This study supports this view. Several of the senior participants spoke about loneliness and “just sitting at home” before the senior group started. The social context and ambience are crucial magnets, and concern not just having the ability to independently move around. Several of the older people also stressed that they would never have done different activities on their own. The social context was important for them; so even if they, in theory, had sufficient mobility resources, they assumed that they would not have undertook the activities by themselves in practice. Again, mobility is more than the physical movement from point A to B (Cresswell 2006).

However, social participation in the neighbourhood must not be restricted to neighbourhood mobility. Rather, it should also involve activities outside the neighbourhood and across other areas. In the current study, this was something shown to be very successful and also much appreciated by the senior participants. What is more, activities beyond the neighbourhood can also encourage a wider range of movement among older people, which is highly relevant for controlling one’s life, as it also can be regarded as positive in light of the desire for the age-friendly city (WHO 2007) and for individuals to independently, safely and comfortably be able to age in place (CDC 2017).

Moreover, the ethnographic method in this case study offered the opportunity to do an in-depth study with a context-dependent perspective. By using this method, I had the opportunity to get to know the neighbourhood from the inside. I also had the opportunity to follow the project and the older people over time as well as ‘travelling along’ with them during activities outside the neighbourhood and observe the phenomenon in motion. Through this method, I was able to gain in-depth knowledge of meaning and context, and capture experiences in real time. In addition, following the senior participants and the project over a period of time gave continuous validation of the empirical material by having the possibility to check with the informants to ensure there were no misunderstandings or misclassifications.

One of the limitations of the study, however, is that it only captures the experiences and reflections of relatively active persons, that is, the older people who participated in the project. As a consequence, further mobility studies in urban neighbourhoods that encompass a wider scope of the meaning of social participation for daily mobility are needed. In this study, social participation had positive effects on the individuals’ daily mobility. However, issues that concern social participation and mobility require more follow-up for future decision-making and implementation.

To summarise, it has been found that stakeholders and staff working closely with older people benefit immensely from genuine engagement as well as from employing a democratic approach: meaning working *with* the older people rather than *for* them. In addition, thinking out of the box with cross-cultural and intergenerational initiatives has, in this study, been shown to be successful, in that it has a broader reach for inclusion and social participation. Although projects related to securing the right to access the city and the daily mobility of older persons are to be commended, in and of themselves, they are insufficient. Instead, they must be an integrated part of urban policies and city planning as a whole. This also points to the need to identify the daily mobility requirements of older persons as a key dimension of not only social sustainability but also of the right of access to the city for all.

## Footnotes

1 The neighbourhood meeting place is open every day, and residents can come in for a free cup of coffee, borrow a computer, borrow the telephone, access the internet, etc.

2 One of the workers at the meeting place.
